# Bioinspired Microhinged Actuators for Active Mechanism‐Based Metamaterials

**DOI:** 10.1002/advs.202407231

**Published:** 2024-11-18

**Authors:** Zi‐Yi Cao, Huayang Sai, Weiwei Wang, Kai‐Cheng Yang, Linlin Wang, Pengyu Lv, Huiling Duan, Tian‐Yun Huang

**Affiliations:** ^1^ Department of Advanced Manufacturing and Robotics State Key Laboratory for Turbulence and Complex Systems BIC‐ESAT College of Engineering Peking University Beijing 100871 China; ^2^ National Key Laboratory of Advanced Micro and Nano Manufacture Technology Beijing 100871 China

**Keywords:** bioinspired microhinge, compliant mechanism, mechanism‐based metamaterials, shape‐morphing, two‐photon direct laser writing

## Abstract

Mechanism‐based metamaterials, comprising rigid elements interconnected by flexible hinges, possess the potential to develop intelligent micromachines with programmable motility and morphology. However, the absence of efficient microactuators has constrained the ability to achieve multimodal locomotion and active shape‐morphing behaviors at the micro and nanoscale. In this study, inspiration from the flight mechanisms of tiny insects is drawn to develop a biomimetic microhinged actuator by integrating compliant mechanisms with soft hydrogel muscle. A Pseudo‐Rigid‐Body mechanical model is introduced to analyze structural deformation, demonstrating that this hydrogel‐based microactuator can undergo significant folding while maintaining high structural stiffness. Furthermore, multiple microhinged actuators are combined to facilitate folding in multiple degrees of freedom and arbitrary directions. Fabricated by a multi‐step four‐dimensional (4D) direct laser writing technique, the microhinged actuators are integrated into 2D and 3D metamaterials enabling programable shape morphing. Additionally, micro‐kirigami with photonic structures is demonstrated to show the pattern transforming actuated by the microhinges. This bioinspired design approach opens new avenues for the development of active mechanism‐based metamaterials capable of intricate shape‐morphing behaviors.

## Graphical abstract

Drawing inspiration from the hinge mechanism of insect wings, a microhinged actuator is reported. Unlike traditional bi‐layers, this biomimetic design integrating soft artificial muscles and compliant mechanisms can achieve more significant folding deformation and higher structural stiffness. Furthermore, the microhinge designs are expanded for multi‐orientation and multi‐degree‐of‐freedom folding, allowing for the complex shape morphing of mechanism‐based metamaterials at the microscale.

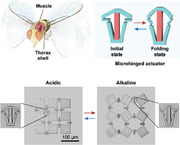

## 1 Introduction

Mechanism‐based metamaterials are a distinct class of shape‐morphing metamaterials found in complex mechanical systems.^[^ ^1^, ^2^, ^3^ ^]^ These metamaterials can transform into various structural forms by employing rigid skeleton frameworks and flexible articulations, resembling systems such as the natural musculoskeletal system or reconfigurable tensegrity system. By programming the functional units within their systems, these rigid‐flexible coupled mechanical metamaterials can switch between shapes with unconventional physical properties, such as negative thermal expansion,^[^ ^4^ ^]^ negative refractive index,^[^ ^5^ ^]^ and invisibility cloaking.^[^ ^6^ ^]^ Furthermore, the stimuli‐responsive properties of smart materials, such as hydrogels,^[^ ^7^, ^8^, ^9^, ^10^, ^11^ ^]^ liquid crystal elastomers (LCEs),^[^ ^12^, ^13^, ^14^, ^15^ ^]^ and shape memory polymers (SMPs),^[^ ^16^, ^17^, ^18^ ^]^ enable these metamaterials to adapt to environmental conditions, facilitating active shape transformations across multiple configurations. These active shape‐morphing metamaterials have attracted considerable attention for applications in flexible electronics,^[^ ^19^, ^20^, ^21^ ^]^ biomedical devices,^[^ ^22^ ^]^ autonomous robotics,^[^ ^23^, ^24^ ^]^ deployable antenna,^[^ ^25^ ^]^ and optical microdevices.^[^ ^26^ ^]^

The development of active mechanism‐based metamaterials necessitates the design of flexible microhinges within constrained spatial dimensions to efficiently manipulate rigid skeleton structures.^[^ ^27^ ^]^ One of the main challenges is to produce flexible microhinged actuators that can exert substantial force and displacement outputs. Unlike macroscale machines, microactuators require high integration of sensing, actuation, and mechanical systems into compact bodies. Additionally, at the microscale, issues such as multiple component assembly, limited fabrication technologies, and increased surface forces, pose difficulties in microhinged actuator design. Typically, shape‐morphing behavior in metamaterials is achieved by precisely tuning the strain mismatch between multiple layers of films or beams.^[^ ^28^, ^29^, ^30^, ^31^ ^]^ However, this method poses limitations such as asynchronous responsiveness of different materials, mismatching in thickness and stiffness between layers, and failure in interface bonding, all of which constrain the achievable deformations. Four‐dimensional (4D) direct laser writing based on two‐photon polymerization has been developed to address these limitations.^[^ ^32^ ^]^ Using this advanced microprinting technique with submicron precision, mechanical advantage is introduced to significantly enhance shape‐morphing capabilities by printing microscale passive hinges with active heterogeneous bi‐layered beams. However, increasing deformation by reducing the layer thickness produces relatively low hinge torque which hinders the morphological transformation of large‐scale metamaterials.^[^ ^33^ ^]^ Additionally, this one‐step fabrication strategy also prints bi‐layered microactuators using a single photopolymerized material with nonlinear crosslinking gradients via an adjustable dose laser. The adjustable range of heterogeneity between different layers is narrow, and the stringent material selectivity imposes significant restrictions on potential applications.

To break through the single material constraint, diverse materials need to be precisely programmed into rigid‐flexible coupled structures of active metamaterials. Drawing inspiration from nature, the musculoskeletal system exemplifies a rigid‐flexible coupled morphable structure that facilitates complex 3D motion. Even tiny flying insects, such as Drosophila, which measure as small as 400 µm,^[^ ^34^ ^]^ have evolved efficient wing hinge mechanisms within their thorax. These mechanisms involve specialized thoracic skeletal structures and interconnected flight muscles that induce thoracic vibrations.^[^ ^35^ ^]^ Amplified by the skeletal mechanisms, minute muscle contractions result in significant and powerful wing folding, allowing for remarkable stability and nimble maneuverability at high speeds of flight. This compact musculoskeletal design serves as a model for developing bioinspired microscale actuators for active mechanism‐based metamaterials.

In this paper, we introduce a multi‐material microhinged actuator inspired by insect wing hinges. Employing the multi‐step direct laser writing, a compliant skeleton mechanism with high stiffness and soft hydrogel muscles with environmental responsiveness is integrated into the mechanism‐based metamaterial. Through structural analysis and parameter optimization, compliant mechanisms are designed to enable microhinged actuators with substantial folding deformation and high structural stiffness. These initial microhinges are then modularly assembled to create advanced microhinges with multi‐orientation folding and multi‐degree‐of‐freedom (DOF) folding deformations. Further, these microhinges are integrated into kirigami and network metamaterials. This deformation strategy enables the realization of micro‐kirigami with 2D in‐plane and out‐of‐plane morphing, as well as micro‐networks with 2D and 3D anisotropic shape transformations. Finally, to demonstrate the potential applicability of this strategy, photonic structures are programmed onto the micro‐kirigami to achieve active pattern transformation. The resulting shape‐morphing metamaterials, incorporating both soft and rigid components, offer significant potential for integrating functional materials and advancing active mechanism‐based metamaterials.

## 2 Results

### 2.1 Inspiration and Design

**Figure 1:**
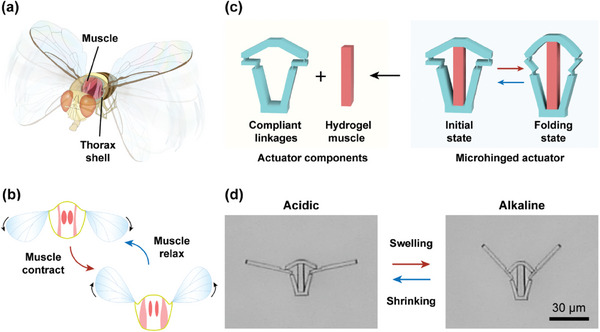
Schematics of bioinspired microhinged actuators. a) Schematic diagram of the musculoskeletal system of a flying insect. b) Deformation mechanism of the flight system. c) Design flow of actuator components and shape‐morphing performance of artificial microhinged actuators. d) Optical images of the microhinged actuators in the initial (acidic) and deformed (alkaline) states.

In nature, tiny flying insects have evolved highly sophisticated flight mechanisms that enable rapid and efficient wing folding, allowing them to evade predators and locate food sources. Researchers have studied the anatomy of insect wings in detail to better understand their flight capabilities.^[^ ^34^ ^]^ A key component of their wing‐folding ability is the specialized linkage system within their narrow thorax shells, as depicted in **Figure** **1**a. By utilizing dorsoventral and dorsolateral muscles attached to the upper plate of the thorax, as shown in Figure 1b, flying insects can indirectly regulate the kinematics and dynamics of their wings. Through a leverage mechanism, these short links convert muscle contractions and expansions into significant wing folding. Furthermore, the symmetrical linkage system ensures coordinated movement of the wings. This compact and efficient arrangement of muscular and skeletal structures offers a promising model for developing actuators with shape‐morphing behaviors, especially at the microscales.

In this study, a novel microhinged actuator inspired by insect wings is introduced to enable substantial and powerful shape morphing. These microhinged actuators consist of two materials: compliant linkages and active pH‐responsive hydrogel blocks, as illustrated in Figure 1c. The linkages are connected via compliant thin joints with a thickness of 400 nm and a length of 1.5 µm. The reduced joint thickness facilitates greater deformation, as the modulus of the linkage material (IP‐L 780 commercial photoresist, Nanoscribe GmbH) is significantly higher than that of the hydrogel. When the environmental pH exceeds 8.5, the electrostatic repulsion between similarly charged COO─ groups in the polymer network increases the hydration of the hydrogels, causing the hydrogel to swell by a ratio of 1.3. The swollen hydrogel then actuates the compliant linkage to deform. The folding deformation is achieved through the rigid rotation of the symmetric linkage, as depicted in Figure 1d. Conversely, when the environmental pH falls below 8.5, the hydrogel shrinks as the electrostatic repulsion between the uncharged COOH groups dissipates. The hydrogel will suddenly and completely swell or shrink at pH ≈8.5. Therefore, acidic and alkaline conditions are used to measure the structural deformation when hydrogels fully shrink or swell.

### 2.2 Single Microhinged Actuator

#### 2.2.1 Mechanical Model

**Figure 2:**
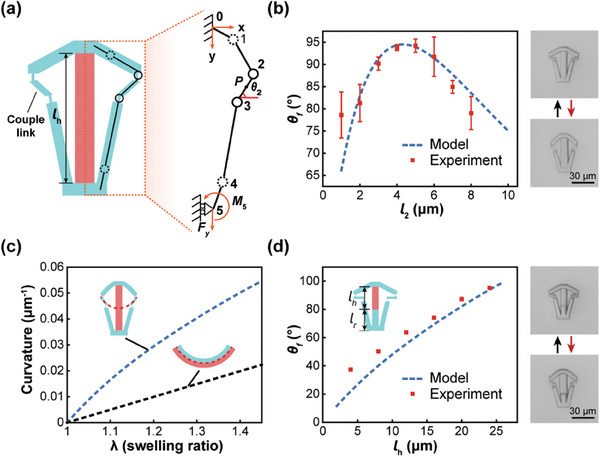
Influence of linkage geometrical parameters on deformation. a) Mechanical model of a single microhinged actuator. b) Influence of the length of couple links *l*_2_ on the folding angle *θ*_f_. c) Comparison of bending curvature of microhinged actuator and bilayer. d) Influence of hydrogel length *l*_h_ on the folding angle *θ*_f_ of the optimal microhinged actuators.

Understanding the deformation kinematics of micro‐hinged actuators is essential for optimizing their structural design and functional performance. A theoretical framework based on the Pseudo‐Rigid‐Body Model was applied to quantitatively assess the deformation of the compliant linkage (as detailed in Supporting Information).^[^ ^37^ ^]^ The mechanical model for a single microhinged actuator is illustrated in **Figure** **2a**. To simplify the analysis, only the right half of the structure was considered due to the symmetrical nature of the IP‐L‐based mechanism. In this model, the thicker bars of the linkage are assumed to act as rigid bodies undergoing rotation, while the thinner joints are modeled as torsion springs. The hydrogel block is assumed to be a linear elastomer. Upon swelling, the hydrogel, constrained by the compliant linkages, experiences compressive forces along the y‐axis, leading to compressive deformation while maintaining force equilibrium

(1)

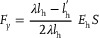

where *l*_h_ and *l*_h_′ represent the initial and deformed lengths of the hydrogel block, respectively, *λ* is the free swelling ratio, *E*_h,_ and *S* denote Young's Modulus and cross‐sectional area of the hydrogel, respectively.

The compliant linkage is subjected to a pair of opposing forces, maintaining the moment equilibrium, as given by

(2) M+K·Δθ=F·L

where ***M*** is the moment applied to the linkage, ***F*** represents the force acting on the linkage, Δ ***θ*** is the rotation angle of bars in the linkage, and ***L*** is an equivalent length vector of the linkages (see Section S1 Supporting Information).

Furthermore, the deformed linkage and hydrogel must satisfy the geometric coordination expressed by

(3) $$l_{h}^{\prime }=\sum_{0}^{5}l_{i}\sin\theta_{i}^{\prime }+\frac{l_{j}}{2}A\left(\theta_{i}\right)$$

where *l_i_* and *θ_i_* ′ are the lengths and deformed angles of bar *i*, *l*_j_ denotes the length of the joint, *A*(*θ_i_*) is a shape‐dependent coefficient. The subscript *i* refers to the bar numbers, which are labeled in detail in Figure S1 (Supporting Information).

The angular displacement of each bar in the compliant linkage can be obtained by solving Equations ((1), (2) and 3). Newton's method is employed to numerically solve these equations using MATLAB software to obtain the folding angle. The length of the joint is fixed at *l_j_* = 1.5 µm, and the overall geometry of the linkage is determined by five independent variables: the coordinates of the center point *P*, the length and initial angle of the couple link (*x_P_, y_P_, l*_2_ *, θ*_2_) and the initial length of the hydrogel *l*_h_. Once these variables are established, other geometric parameters can be deduced based on the corresponding geometric relationships (as detailed in Supporting Information). Given that the folding angle of microhinged actuators *θ*_f_ = 2(*θ*_2_′−*θ*_2_) is governed by these five variables, their effects on the folding angle are further discussed in the subsequent section.

#### 2.2.2 Parameters Analysis

To optimize the configuration of the IP‐L linkage for maximizing the folding angle, the effects of the linkage geometry parameters (*x_P_, y_P_, l*_2_ *, θ*_2_) on folding angle *θ*_f_ were examined, while maintaining a constant hydrogel length *l*_h_ = 24 µm, as shown in Figure S2 (Supporting Information). The analysis reveals that larger folding angles are achieved by minimizing the values of *x_P_*, *y_P_*, and *θ*_2_. Additionally, the theoretical results indicate that the relationship between the couple link length *l*_2_ and the folding angle *θ*_f_ is non‐monotonic. Taking into account the design and fabrication constraints related to printing precision and alignment accuracy, the influence of *l*_2_ on the folding angle was explored by fixing *x_P_* = 9.5 µm, *y_P_* = 6.35 µm, and *θ*_2_ = 0°, as illustrated in Figure 2b. The theoretical model predicts a maximum folding angle of 93.92° when *l*_2_ is set as 4.2 µm.

Optimal microhinged actuators were fabricated using sequential two‐photon direct laser writing to validate the theoretical predictions. The initial and deformed states of the optimal microhinged actuator are depicted in Figure 2b, showing a folding angle of ≈90°, consistent with the theoretical model. The bending curvatures of the microhinged actuators are compared with bilayers made of the same materials, as shown in Figure 2c, with varying swelling ratio *λ*. The bending curvature of the microhinged actuator is at least two times larger than that of the bilayer when *λ* = 1.3.

Besides, the bending stiffness of the microhinged actuators was compared with that of bilayers exhibiting the same bending curvature. The bending stiffness of microhinges was obtained through finite element analysis (FEA), while the bending stiffness of bilayers was calculated using elastic mechanics theory. FEA and theoretic results demonstrate that the bending stiffness of microhinged actuators (19 170 N·µm^2^) is substantially greater than that of bilayers (38 N·µm^2^) (see Section S4, Supporting Information). This indicates that microhinged actuators are more stable under load and capable of generating larger output torque than bilayers.

Microhinged actuators with adjustable folding angles are requested for achieving programmable shape morphing. Among various methods for controlling the folding angle, reducing the hydrogel block length *l*_h_ provides a straightforward and effective approach, which involves substituting the reduced hydrogel length *l*_r_ with IP‐L resin, as shown in Figure 2d. The relationship between *l*_r_ and *l*_h_ is given by *l*_r_ + *l*_h_ = 24 µm. In Figure 2d, the experimental results are compared with the theoretical results of the folding angles for varying lengths of the hydrogel block. Experimental data indicate that when the hydrogel length is adjusted from 4 to 24 µm, the folding angle can be tuned from 37.5° to 95.3°.

The joint width *w*_j_ in the IP‐L linkages is another critical factor affecting the linkage stiffness and folding angle. As indicated by the relationship between *w*_j_ and the stiffness constant *K* (described in Equation S1, Supporting Information), increasing the joint width enhances the linkage stiffness, thereby reducing the folding angle. To illustrate this, tests were conducted on microhinged actuators with joint widths of 400, 800, and 1000 nm, as depicted in Figure S3 (Supporting Information). A joint width of 400 nm was selected as the design parameter to enable significant deformation while ensuring high printing accuracy, as shown in the close‐up scanning electron microscope (SEM) images of Figure S4 (Supporting Information).

The fatigue resistance of the microhinged actuators was evaluated by subjecting them to multiple pH cycling tests, as thin joints are prone to damage during deformation. Figure S5 (Supporting Information) illustrates the performance of the actuators after repeated deformation cycles. Even after 15 pH change cycles, the folding angle remained at ≈80°, indicating that the thin joints remained undamaged during repeated deformations.

### 2.3 Optimal Designs of Microhinged Actuators

**Figure 3:**
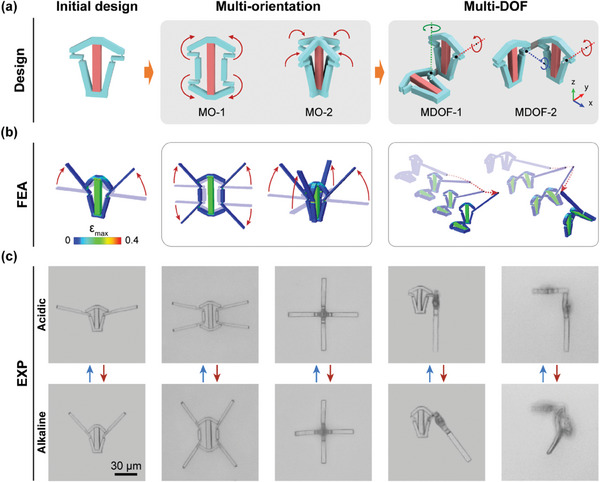
Design and FEA simulation of bioinspired microhinged actuators. a) Schematics of the microhinged actuator designs showing multi‐orientation and multi‐DOF in folding deformations. b) Simulation results showing the deformation of bioinspired microhinged actuators. c) Optical images displaying the deformation of bioinspired microhinged actuators.

Metamaterials in both 2D and 3D often exhibit unique functions and adaptable behaviors through intricate local folding deformations. In this study, microhinged actuators were designed to fold in multiple orientations and degrees of freedom (DOF) by adjusting or assembling the initial microhinged structures, as depicted in **Figure** **3**.

To achieve multi‐orientation folding, we increased the number of coupled links in compliant linkages. Figure 3a presents a 2D microhinge design with bi‐directional folding deformation along a single axis and a 3D microhinge design with bi‐directional folding deformation across multiple axes. In these multi‐orientation folding microhinges, the rotation of various connectors is controlled by a single hydrogel block, ensuring synchronous and coordinated deformation.

For multi‐DOF folding microhinges, we assembled microhinges with different configurations. This allowed us to achieve folding deformations around multiple axes by superimposing the deformations of microhinges arranged in various planes, as shown in Figure 3a. The assembled multi‐DOF microhinges demonstrated simultaneous rotation around the *x*/*z* and *x*/*y* axes. FEA models were employed to predict these deformations. Additionally, extension bars were attached to the coupling links as connectors, allowing for a clearer visualization of the folding deformations. As depicted in Figure 3b, when hydrogel blocks swell, these multi‐DOF microhinges enable complex 3D trajectories.

The microhinges were fabricated using sequential two‐photon printing and activated by changing the environmental pH to validate the feasibility of the aforementioned designs. As shown in Figure 3c, the deformation of these microhinges closely aligns with the FEA predictions, demonstrating the predictable behavior of these optimal microhinges. The 3D microhinges (MO‐2, MDOF‐1, and MDOF‐2) are photographed from the top view to present their deformations better.

### 2.4 Fabrication Method

Microhinged actuators are integrated into metamaterials to enable active shape morphing. The metamaterial samples, composed of two different materials, are fabricated through a sequential two‐photon printing process. A typical kirigami structure was employed to demonstrate the feasibility of both the manufacturing process and the deformation strategy. By incorporating microhinged actuators at the deforming points in the kirigami structure, precise large‐scale folding deformations can be achieved in localized areas.

**Figure 4:**
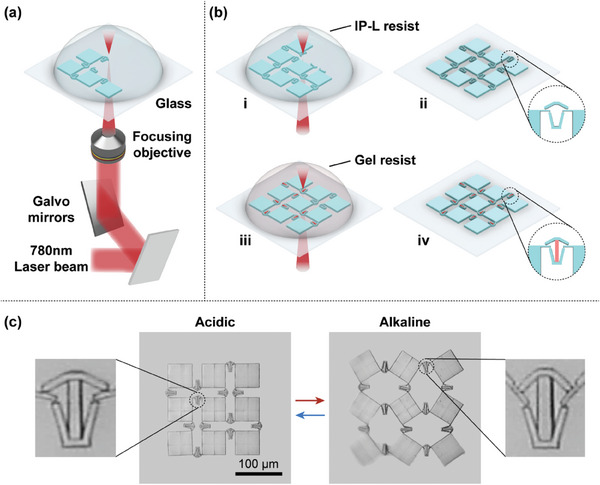
The fabrication process of micro‐kirigami with microhinged actuators. a) Schematic of two‐photon polymerization direct laser writing. b) Schematics of the micro‐kirigami fabrication process using sequential two‐photon printing: i, ii) First cross‐linking and development of IP‐L structures. iii, iv) The second printing step for hydrogel muscles. b) Optical images of micro‐kirigami in the initial (acidic) and deformed (alkaline) states.

The manufacturing process is illustrated in **Figure** **4**a,b. A laser beam with a central wavelength of 780 nm, a pulse duration of 100 fs, and a repetition rate of 80 MHz is focused onto the photoresist using an objective lens. The focused laser spot is scanned in 3D by controlling two galvano mirrors and a piezo stage, as shown in Figure 4a. The compliant linkages of the microhinged actuators and rigid plates of the kirigami structure are printed together in the first step, using IP‐L photoresist under a 63× objective in oil immersion mode. The unexposed resist is then removed with isopropyl alcohol (IPA) for 10 min, and the IP‐L structures are dried. Subsequently, the IP‐L structures are submerged in a hydrogel precursor solution, where hydrogel blocks are printed and cross‐linked with the IP‐L structure. Afterward, the structures are rinsed with IPA for 15 min to remove the remaining hydrogel precursor solution. Before printing the hydrogel blocks, the printing stage must be accurately aligned with the IP‐L structure within the direct laser writing system. During the second printing step, it is necessary to print repeatedly at the interface of the hydrogels and IP‐L structures to ensure a secure connection between the hydrogel blocks and the IP‐L linkages with a thickness of 2 µm. The swelling and mechanical properties of the hydrogels can be modified by adjusting the laser power. A higher laser power results in a denser hydrogel network with a higher modulus, but a lower swelling ratio. For this study, a laser power of 40 mW was selected for hydrogel printing to ensure sufficient driving force and deformation. Finally, the samples are immersed in dilute hydrochloric acid before testing. To simulate the microhinged actuators, an appropriate amount of NaOH solution is added to the dilute hydrochloric acid. Due to the small size of the hydrogels in the microhinged actuators, the solution diffuses more rapidly through the network compared to larger‐scale hydrogel structures, enabling faster deformation. The response time of a single microhinge was recorded when exposed to NaOH solutions of varying concentrations. As shown in Figure S6 (Supporting Information), higher NaOH concentrations accelerate the deformation process, with the shortest response time being less than 5 s.

Close‐up SEM images of the two‐material interfaces reveal that the hydrogel muscle is tightly attached to the IP‐L compliant mechanism before deformation, as shown in Figure S7 (Supporting Information). Furthermore, as shown in Figure S5 (Supporting Information), the good performance of the microhinged actuators after repeated deformation proves the close connection between the two materials during and after deformation. This robust connection is crucial for enabling the hydrogel to drive the deformation process. The kirigami structure's ability to deform was demonstrated by altering the pH of the surrounding liquid solutions. As the hydrogels swell and contract, the microhinged actuators in various regions act in unison to deform and rotate the rigid plate, as depicted in Figure 4b. Metamaterials actuated by these microhinged actuators exhibit significant and predictable shape‐morphing capabilities.

### 2.5 Metamaterials with Microhinged Actuators

**Figure 5:**
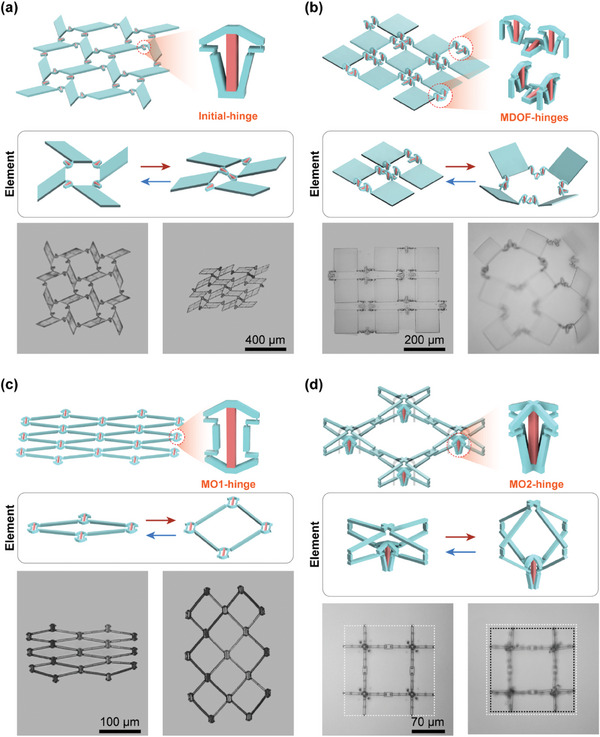
Experimental results of active mechanism‐based metamaterials with bioinspired microhinged actuators. Schematic and optical images of a) the micro‐kirigami with swelling‐shearable behavior, b) the micro‐kirigami with out‐of‐plane swelling behavior, c) the 2D micro‐network with anisotropic behavior, and d) the 3D micro‐network with anisotropic behavior.

The ability of mechanism‐based metamaterials to undergo shape transformation is essential for applications, such as flexible electronics and implantable medical devices. In this study, several metamaterials capable of diverse deformations were designed using microhinged actuators. These metamaterials were constructed based on kirigami and network designs, incorporating active hinges and rigid plates or bars, as shown in **Figure** **5**.

Kirigami presents a promising approach for creating materials that can undergo various deformations by altering the shape of thin sheets or films. Typically, kirigami structures deform in response to mechanical forces. However, this study explores stimuli‐responsive kirigami‐based metamaterials activated by microhinges. The kirigami structure developed in Section 2.4, comprising rigid plates and initial microhinges, achieved isotropic swelling deformation. In this design, 2D square plates are connected by microhinges positioned at pivot points in different orientations. The geometry of the rigid plates and the hinges plays a key role in determining the deformation behavior of kirigami‐based structures.

By modifying the shape of the plates, different deformation modes, such as shearing, can be achieved, as illustrated in Figure 5a. The rigid plates are arranged in a parallelogram configuration, with two sides designed as 60 and 120 µm in length, and internal angle of 45°. The microhinges are oriented at ± 45° and ± 135° relative to the horizontal axis. When the environmental pH changes, the microhinges induce rotation of the rigid plates, resulting in active deformation that resembles shear within the micro‐kirigami structure. Experimental results indicate that the shear angle reaches ≈25°, determined by the geometry of the parallelogram‐shaped rigid plates.

Additionally, micro‐kirigami with out‐of‐plane deformation was fabricated by incorporating multi‐DOF microhinges, as shown in Figure 5b. Two microhinges folded around the y‐axis were integrated into the initial microhinges to preserve structural symmetry. As the pH level changes, these multi‐DOF microhinges actuate the rigid plates to rotate, producing simultaneous out‐of‐plane deformation. By adjusting the microhinges and the shape of the elements, more intricate kirigami‐based metamaterials can be developed. The proposed microhinged actuators provide a new method for actuating microscale kirigami‐based metamaterials.

Metamaterials often feature network geometries, such as lattice structures. Both 2D and 3D metamaterials have demonstrated shape‐morphing capabilities using multi‐orientation microhinges. Figure 5c shows a 2D micro‐network capable of anisotropic shape transformation. The network consists of four IP‐L bars (80 µm in length, 5 µm in width) and four 2D multi‐orientation microhinges. When the pH value changes from acidic to alkaline, the micro‐network expands anisotropically, with a 259% increase along the *y*‐axis and a 73.8% increase along the *x*‐axis.

This 2D anisotropic shape morphing was further extended to a 3D micro‐network. In Figure 5d, the 3D network element consists of four bars, each 40 µm in length and 3 µm in width, connected by five passive thin joints (2.5 µm length, 0.8 µm width) and a 3D multi‐orientation microhinge. When the pH value changes, the 3D micro‐network exhibited swelling ratios of ≈92.8% along the *x*‐and *y*‐axes, and 133.3% along the *z*‐axis. Experimental images of the initial and deformed states from a top‐down view are presented in Figure 5d. When printed vertically, some parts of the microhinges overhang during the printing process, leading to potential structural collapse. To mitigate this, geometrical parameters were adjusted by increasing the thickness and length of the thin joints from 400 nm and 1.5 µm to 800 nm and 2.5 µm, respectively. In addition, the scan speed was increased from 8000 to 9000 µm ^−1^s, and the laser power was raised from 40 to 50 mW to further reduce the risk of collapse. Overall, microhinged actuators with various folding deformations offer a robust means of constructing microscale metamaterials with complex shape‐morphing capabilities.

### 2.6 Shape Morphing for Photonic Metamaterial

**Figure 6:**
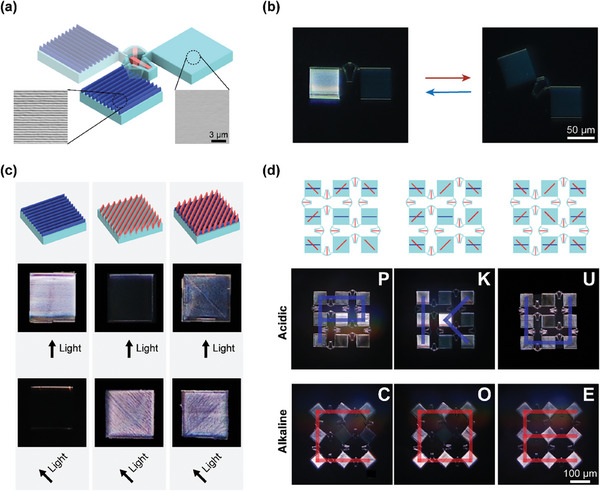
Shape morphing of micro‐photonic metamaterials integrated with microhinged actuators. a) The kirigami unit with photonic structures. b) Shape morphing of the photonic unit. c) Photonic structures at 0° (blue) and 45° (red) and their superposition form. d) pH‐responsive pattern transforming of pre‐programmed photonic kirigami.

The multi‐material composition of these shape‐morphing microstructures enables their integration with various functional devices and thus has broadened application prospects. One of the potential applications is optical metasurfaces. Changes in the shape or orientation of photonic structures have been used to change the structural color, thereby encrypting, storing, hiding information, etc.^[^ ^38^, ^39^, ^40^ ^]^ In this regard, we integrated microhinged actuators into micro‐kirigami with photonic structures to achieve environmentally responsive optical pattern transformations. A kirigami unit is utilized to illustrate the principle of structural color change, as shown in **Figure** **6**a. The photonic structures are directly printed onto the kirigami blocks using two‐photon direct laser writing, with a height of 1 µm and a width of 700 nm. When the direction of light is perpendicular to the photonic structure, it results in maximum light reflection; otherwise, the reflection is weakened. Therefore, when the microhinged actuator deforms, the grating block rotates and darkens as the angle between the grating and the light source changes, as shown in Figure 6b. Further, these photonic structures are programed and integrated into micro‐kirigami driven by microhinged actuators. In order to encode multiple information into the same kirigami structure, we introduced photonic structures in different directions and their superposition forms, as shown in the blue and red parts in Figure 6c. When the kirigami deforms, the rotate angle of blocks changes from 0° to ± 45°. The photonic structures are programmed at 0° and ± 45° with “PKU” and “COE” patterns into the 3 × 3 kirigami structures. When the environment pH changes, the kirigami units rotate actuated by the microhinges, and the pattern changes from “PKU” to “COE”, as shown in Figure 6d. The active shape‐morphing strategy we proposed is compatible with the multi‐material functions at the microscale, with potential application in fields such as optical information encryption, providing a new approach for the next generation of miniaturized smart devices.

## 3 Discussion

This study presents microhinged actuators, inspired by insect wing hinges, designed to facilitate active shape‐morphing in mechanism‐based metamaterials. These hydrogel‐based microhinged actuators enable a wide range of adjustable folding deformations while maintaining high structural stiffness. Additionally, we have developed microhinges that support multi‐orientation and multi‐DOF folding deformations. Leveraging the versatility and efficiency of these microhinges, we fabricated programmable shape‐morphing 2D and 3D kirigami and network micro‐metamaterials. The resultant micro‐kirigami have been used as carriers of photonic structures to achieve environmentally responsive pattern transformations. These microscale mechanism‐based metamaterials exhibit significant shape‐morphing capabilities despite being constructed primarily from highly rigid materials.

Our proposed actuation methodology introduces a new approach to multi‐material active shape‐morphing devices. The rigid parts, made of IP‐L 780 commercial photoresist, provide a platform for integrating functional components such as microcircuits and on‐board sensors. Metamaterials activated by microhinges can serve as responsive substrates for electrical and magnetic systems, with potential applications in wearable devices, wireless microrobotics, and tissue engineering. By incorporating different stimulus‐responsive materials within the microhinges, multi‐stimulus‐responsive metamaterials can be achieved. Furthermore, the independence of the microhinge deformations facilitates the individual control of each actuator within the metamaterial. This approach enables the realization of active shape‐morphing devices with multiple functions and wide‐ranging applicability.

## 4 Experimental Section

### Materials

A commercial photoresist (IP‐L 780 resin, Nanoscribe, GmbH) was used to print the passive part of the microhinged actuators.

The active shape‐morphing sections were created using a pH‐responsive hydrogel. The hydrogel precursor was prepared following a typical procedure detailed in previous works.^[^ ^32^, ^33^ ^]^ Functional monomers, N‐isopropyl acrylamide (NIPAAM, 98%), and Acrylic acid (AAc, 99%) were dissolved in 0.2 mL of ethyl lactate (EL, 98%). Additionally, 0.2 g of polyvinyl pyrrolidone (PVP, average Mw ≈1 300 000) was added to the mixture and stirred until fully dissolved. To this, 0.5 mL of triethanolamine (TEA, 99%), 0.4 mL of dipentaerythritol pentaacrylate (DPEPA, 98%), and 100 µL of a 20wt % solution of 4,4′‐bis(diethylamino) benzophenone (EMK, 97%) in N,N‐dimethylformamide (DMF, 99.5%) were added as the photoinitiator. The swelling behavior of the hydrogel can be further optimized by adjusting the material ratio.

### Fabrication of The Multi‐Material Microstructures

The multi‐material microstructures were fabricated using a commercial two‐photon direct laser writing system (Nanoscribe GmbH, Germany). First, 3D models were designed using the SolidWorks software and converted into STL format files, which were then translated into General Writing Language (GWL) via Describe software and uploaded into the Nanoscribe system for printing.

The borosilicate cover glass substrate was cleaned with isopropyl alcohol (IPA) and dried with nitrogen. A drop of IP‐L resist was placed at the center of the glass substrate, which was then put into the printing system using a 63 × /1.4 oil immersion objective (Carl Zeiss AG). The first printing process involved setting the laser power to 40 mW and the scanning speed to 8000 µm ^−1^s to crosslink the IP‐L resin. After the first printing, the sample was immersed in IPA for 5 min to develop and was subsequently dried with nitrogen.

In the second printing step, the pH‐responsive hydrogel precursor was dropped onto the existing structure on the glass substrate, which was then placed back in the Nanoscribe system in the same position as the first printing. Before the second printing, the printing stage was adjusted with the NanoWrite software using the integrated camera to align with the existing structure. During the second printing, the laser power and scanning speed were set to 40 mW and 2000 µm ^−1^s, respectively, to crosslink the pH‐responsive hydrogel. After the second printing, the sample was developed in IPA two times for 8 min and then dried with nitrogen. Before testing, the microstructures were immersed in dilute hydrochloric acid for at least 30 min.

### Characterization

To characterize the deforming process, samples were first immersed in dilute hydrochloric acid to ensure adequate hydrogel shrinkage. Subsequently, the NaOH solution is dropped to trigger the swelling of the hydrogel blocks. The deformation of the fabricated microstructures was monitored and recorded using an optical microscope (RH‐2000, Hirox, Japan).

The samples were dried under nitrogen for SEM images, and then coated with a 5 nm thick gold film. Optical microscopy images were captured using a TESCAN‐MAIA 3 GMU microscope.

## Conflict of Interest

The authors declare no conflict of interest.

## Supporting information



Supporting Information

Supplemental Video 1

Supplemental Video 2

Supplemental Video 3

Supplemental Video 4

Supplemental Video 5

Supplemental Video 6

## Data Availability

The data that support the findings of this study are available from the corresponding author upon reasonable request.
