# Supplementary material for: Bioinspired Microhinged Actuators for Active Mechanism‐Based Metamaterials
Source: Adv Sci (Weinh). 2024 Nov 18;12(2):2407231. doi: 10.1002/advs.202407231 (PMC11727244; doi:10.1002/advs.202407231)
Supplement: Supplementary file 1 — Supporting Information [file ADVS-12-2407231-s007.docx]

Supporting Information

Bioinspired Microhinged Actuators for Active Mechanism-Based Metamaterials

Zi-Yi Cao, Huayang Sai, Weiwei Wang, Kai-Cheng Yang, Linlin Wang, Pengyu Lv, Huiling Duan, and Tian-Yun Huang*

**Section S1.** **Mechanical properties analysis of IP-L and hydrogel**

The mechanical testing microstructures with a shape of the cuboid (200 μm × 200 μm × 50 μm) were fabricated using direct laser writing with IP-L or hydrogel. To characterize the mechanical properties of microstructures, nanoindentation tests were performed at room temperature using Agilent Nano Indenter G200 (Agilent Technologies Inc., Santa Clara, CA, USA) with a Berkovich indentation tip. For each specimen, 9 indents (3 × 3 dot matrix with a 30 μm spacing) were tested to reduces the error caused by factors such as indent position and environmental disturbance. The maximum pressing force is set as 3000 μN and the maximum depth is set as 2 μm. The force-depth curve of the loading-unloading process can be obtained, as shown in the Figure S8. The relationship between the elastic modulus of the indentation point material and the Young’s modulus of the diamond indenter is *E*=(1－*ν*^2^)(1/*E*_r_－(1－*ν*_i_^2^)/*E*_i_)^-1^, in which *ν* and *ν*_i_ are the Poison’s ratio of sample and indenter, *E*_r_ is the reduced modulus calculated from the force-depth curve, *E* and *E*_i_ are the Young’s modulus of the indenter.^[1]^ For Berkovich indentation tip ν_i_=0.07 and *E*_i_=1140 GPa. Since the value of the sample Poisson's ratio has little effect on the calculation results, we assumed that *ν*=1.3. Young’s modulus of IP-L and hydrogel can be obtained as 4.7 GPa and 20 MPa respectively.

**Section S2. Theoretical Analysis on Microhinged Actuator**

The folding deformation of a single microhinged actuator can be described with a simplified model based on the PRBM.^[2]^ The microhinged actuator is composed with an active part (pH responsive hydrogel) and a passive part (IP-L linkages). The IP-L linkage system is consisted of a symmetrical two-part linkage system. The right half of the linkage system is taken for the deformation analysis. As shown in Figure S1, when the microhinged actuator deforms, bar 1 and bar 3 are assumed undergo linear elastic bending deformation while the bar 2 is assumed undergo rigid body rotation. Based on the PRBM, bar 1 and bar 3 are modeled by two rigid bars pinned by a torsional spring (marked as a grey circle in Figure S1). the length of the pseudo-rigid bar is (1­*γ*) *l_i_*. The stiffness of torsional springs *K_i_* is

$K_{i}=\gamma K_{\Theta}\frac{EI_{i}}{l_{i}}$ (S1)

where *I_i_* is the moment of inertia of joint *i* which is defined as *w*_j_*l*_j_^3^/12. To simplify the model, *γ* and *K*_Θ_ are set to be 0.15 and 2.65.^[3]^ The simplified mechanical model is shown in Figure S1. The hydrogel block is regarded as a linear elastomer. Once microhinged actuator deforms, the hydrogel block is subjected to a pair of compressive forces along the y-direction, resulting in a compressive deformation and satisfying the force equilibrium

$F_{y}=\frac{\lambda l_{h}-l_{h}^{'}}{2\lambda l_{h}}E_{h}S$ (S2)

where *l*_h_ and *l*_h_' are the initial length and deformed length of hydrogel block, *λ* is the free swelling ratio, *E*_h_ and *S* representing the Young’s Modulus and cross-sectional area of the hydrogel respectively, do not change with deformation. The passive linkage is subjected to a pair of opposite forces, and satisfies the moment equilibrium, given by

$\boldsymbol{M}+\boldsymbol{K}\cdot\Delta\boldsymbol{\theta}=\boldsymbol{F}\cdot\boldsymbol{L}$ (S3)

where

$\boldsymbol{M}=\left[ \begin{matrix} \begin{matrix} M_{0} & 0 & 0 & 0 \end{matrix} & \begin{matrix} M_{5} & M_{0}+M_{5} \end{matrix} \end{matrix} \right]$

$$\boldsymbol{K}=\left[ \begin{matrix} \begin{matrix} K_{1} \end{matrix} & \begin{matrix} K_{2} & K_{3} \end{matrix} & \begin{matrix} K_{4} \end{matrix} \end{matrix} \right]$$

$$\Delta\boldsymbol{\theta}=\left[ \begin{matrix} \theta_{1}^{'}-\theta_{1} & \theta_{1}^{'}-\theta_{1} & 0 & 0 & 0 & 0 \\ 0 & \left( \theta_{2}^{'}-\theta_{1}^{'} \right)-(\theta_{2}-\theta_{1}) & \left( \theta_{2}-\theta_{1} \right)-(\theta_{2}^{'}-\theta_{1}^{'}) & 0 & 0 & 0 \\ 0 & 0 & \left( \theta_{3}^{'}-\theta_{2}^{'} \right)-\left( \theta_{3}-\theta_{2} \right) & \left( \theta_{3}-\theta_{2} \right)-(\theta_{3}^{'}-\theta_{2}^{'}) & 0 & 0 \\ 0 & 0 & 0 & \theta_{3}^{'}-\theta_{3} & \theta_{3}^{'}-\theta_{3} & 0 \end{matrix} \right]$$

$$\boldsymbol{F}=\left[ \begin{matrix} F_{x} & F_{y} \end{matrix} \right]$$

$$\boldsymbol{L}=\left[ \begin{matrix} {\gamma l}_{1}sin\theta_{1} & (1-\gamma)l_{1}sin\theta_{1}^{'} & l_{2}sin\theta_{2}^{'} & {(1-\gamma)l}_{3}sin\theta_{3}^{'} & \gamma l_{3}sin\theta_{3}^{'} & -l_{h}^{'} \\ {\gamma l}_{1}\cos\theta_{1} & (1-\gamma)l_{1}\cos\theta_{1}^{'} & l_{2}\cos\theta_{2}^{'} & (1-\gamma)l_{3}\cos\theta_{3}^{'} & {\gamma l}_{3}\cos\theta_{3}^{'} & 0 \end{matrix} \right]$$

As labeled in Figure S1, *M_i_*, *F_x_*, and *F_y_* are moments and forces on the linkage, *K_i_* is the stiffness of the thin joints *i*, *θ_i_* and *θ_i_*', are the initial angle and deformed angle of bars in linkage system, *l_i_* is the length of bars in linkage system. In addition, the deformed linkage and hydrogel block satisfy the geometric coordination

$l_{h}^{'}=\sum_{1}^{5} L(1,i)+\frac{l_{j}}{2}A(\theta_{i})$ (S4)

where

$A\left( \theta_{i} \right)=\sin\left( \frac{\theta_{1}^{'}+\theta_{2}^{'}}{2}-\theta_{1}^{'}+\theta_{1} \right)+\sin\left( \frac{\theta_{2}^{'}+\theta_{3}^{'}}{2}-\theta_{3}^{'}+\theta_{3} \right)+\sin\left( \frac{\theta_{1}^{'}+\theta_{2}^{'}}{2}-\theta_{2}^{'}+\theta_{2} \right)$

$+\sin\left( \frac{\theta_{2}^{'}+\theta_{3}^{'}}{2}-\theta_{2}^{'}+\theta_{2} \right)$

*l*_j_ is the length of joint. The angle of each bar in the passive mechanism can be obtained by solving the equilibrium equations (S2, S3, S4).

**Section S3. Theoretical Analysis on Bending Curvature of Bilayer and Microhinged Actuator**

Basing on Timoshenko’s beam theory, bending curvature of bilayers can be expressed as

$\kappa=\frac{1}{h}\times\frac{6\left( \lambda_{2}-\lambda_{1} \right)\left( 1+m \right)^{2}}{3\left( 1+m \right)^{2}+(1+mm_{E})(m^{2}+1/mm_{E})}$ (S5)

where *h* is the length of the bilayer, *m* is the thickness ratio of two layers, *m*_E_ is the Young’s modulus ratio of two layers, *λ_i_* is the swelling ration of two layers. Materials of bilayer are defined as IP-L (Young’s modulus 4.7 GPa; swelling ratio 1) and hydrogel (Young’s modulus 20 MPa; swelling ratio 1.3). Thickness ratio of two layers is defined as 6 to produce larger bending deformation. Length of bilayer *h* is defined as 30 μm.

The bending curvature of the microhinged actuator can be converted from the folding angle *θ*_f_ and its width *w*_h_

$\kappa=\frac{\theta_{f}}{w_{h}}$ (S6)

where the folding angle *θ*_f_ can be obtained by solving the equilibrium equations (S2, S3, S4).

**Section S4. Simulation of the Deformation of Microhinged Actuators**

Commercial software ABAQUS was used to simulate the deformation of microhinged actuators. IP-L part was defined as linear elastic material (Young’s modulus 4.7 GPa; Poison ratio 0.25). The hydrogel part was defined as hyperelastic material, and the mechanical model was imported into ABAQUS with a user-defined subroutine which have been reported in detail in previous works.^[4,5]^

**Section S5. Analysis on Bending Stiffness of Bilayer and Microhinged Actuator**

Basing on theoretical model, the bending curvature of the microhinged actuator *κ*=0.04032 at a swelling ratio of 1.3 for the hydrogel. In this section, bending stiffness of microhinged actuators are compared with bilayers with the same bending curvature^[6]^. The bilayer is set to consist of two layers of hydrogels with different cross-linking densities. Swelling ration of two layers are defined as *λ*_1_=1.3 and *λ*_2_=1.5, and the ratio of Young’s modulus of two layers is 1.053. According to Equation S5, the maximunm bending curvature can be obtained at *m*=1. When curvature of bilayer *κ*=0.04032, its thickness can be obtained as 7.4 μm. The bending stiffness of bilayer

$K_{b}=E_{1}I_{1}+E_{2}I_{2}$ (S7)

where $E_{1}$, $E_{2}$ and $I_{1}$, $I_{2}$ are the Young’s modulus and moment of inertia of two layers. Bending stiffness can be obtained as 3.839×10^-11^ N·m^2^.

Bending stiffness of microhinged actuator is culculated by FEA. 2 steps of analyze have been used. Deformation of the microhinged actuator actuated by hydrogel swelling is simulated in the first step. In the second step, moment boundary conditions are applied at couple links. Moment-folding angle curves are obtained, as shown in Figure S9. The bending stiffness of the microhinge actuator is

$K_{h}=\frac{Mw_{h}}{\Delta\theta_{f}}$ (S8)

where *M* is the moment. Bending stiffness of microhinged actuators can be obtained as 1.917×10^-8^ N·m^2^


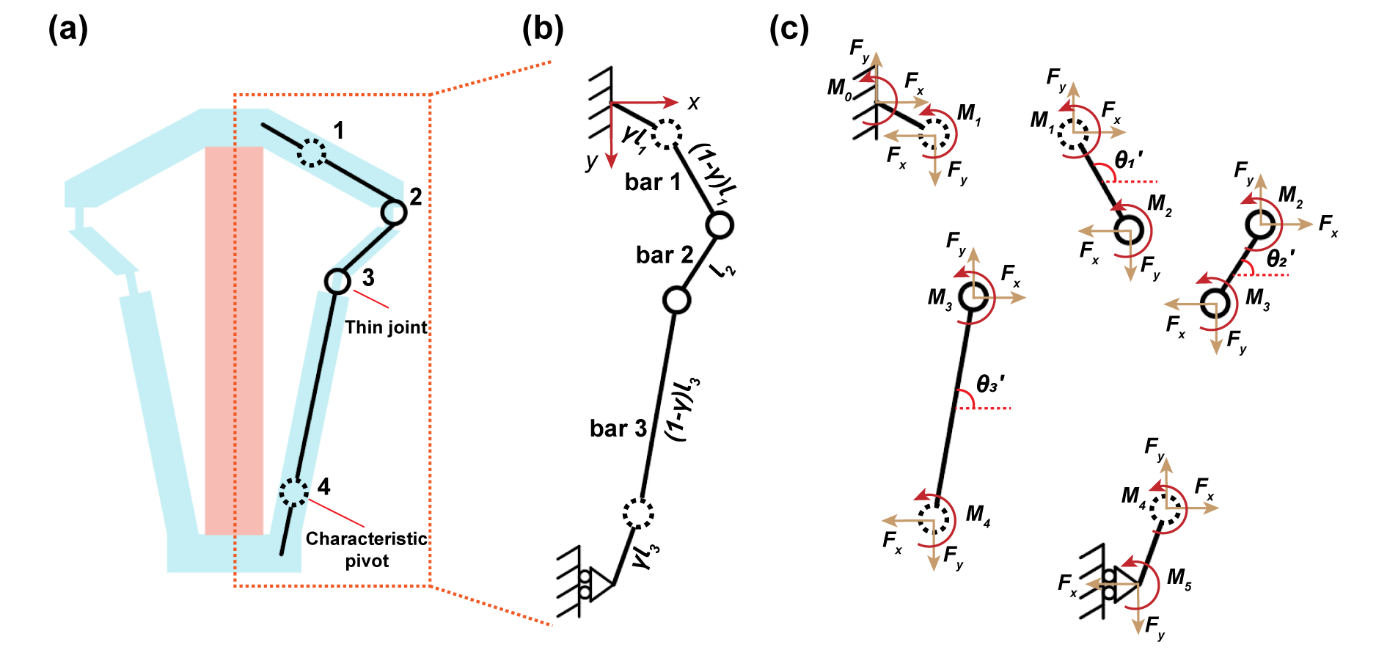


**Figure S1.** Mechanical model of the microhinged actuator. a) Structure schematic of the microhinged actuator. b) The compliant mechanisms are modeled as linkages. c) Force and torques acting on bars.


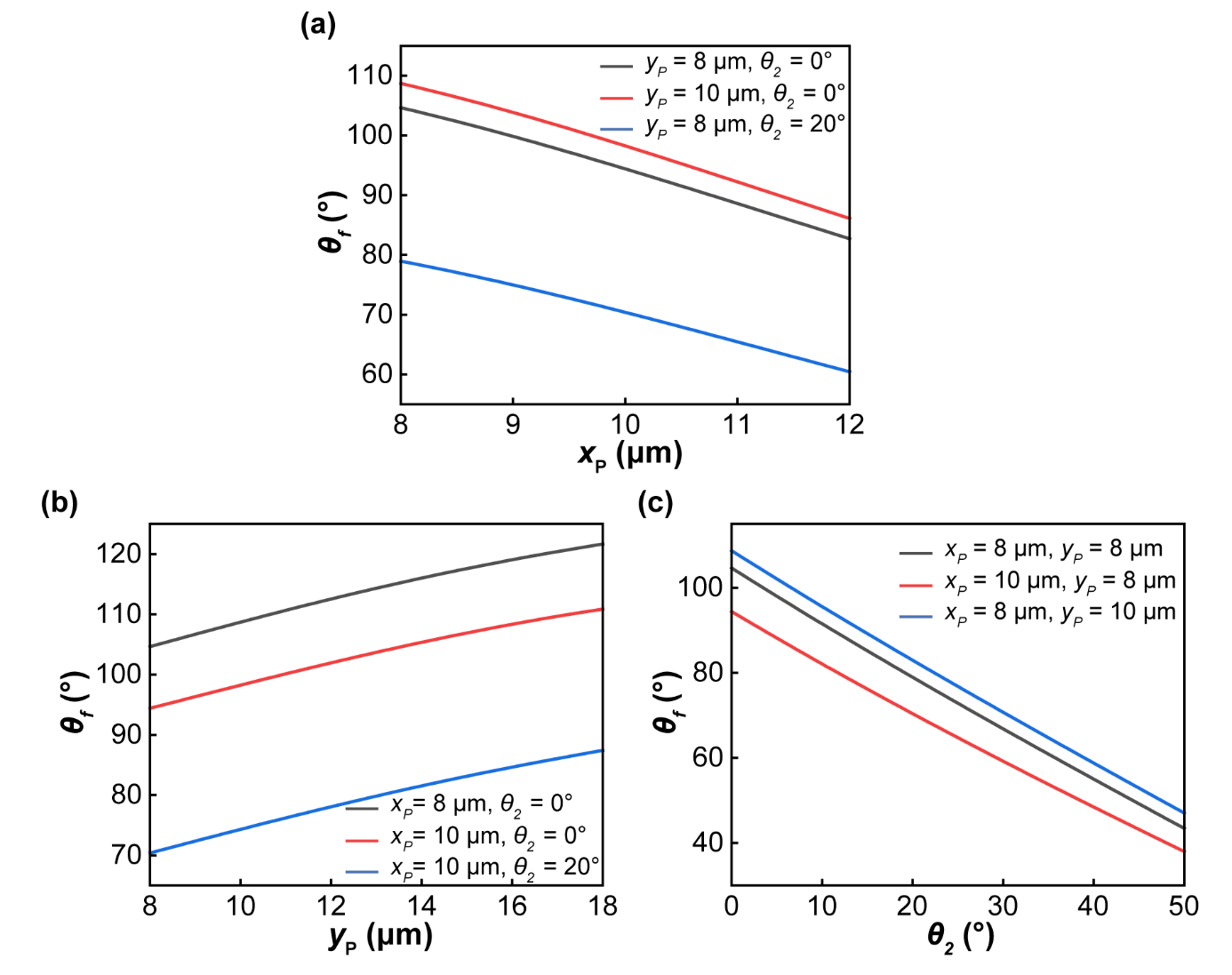


**Figure S2.** Influence of *x*_P_, *y*_P_, and *θ*_2_ on the folding angle of microhinged actuator when fixing *l*_h_=24 μm and *l*_2_=5 μm. a-c) Calculated folding angles for varying *x*_P_, *y*_P_, and *θ*_2_.


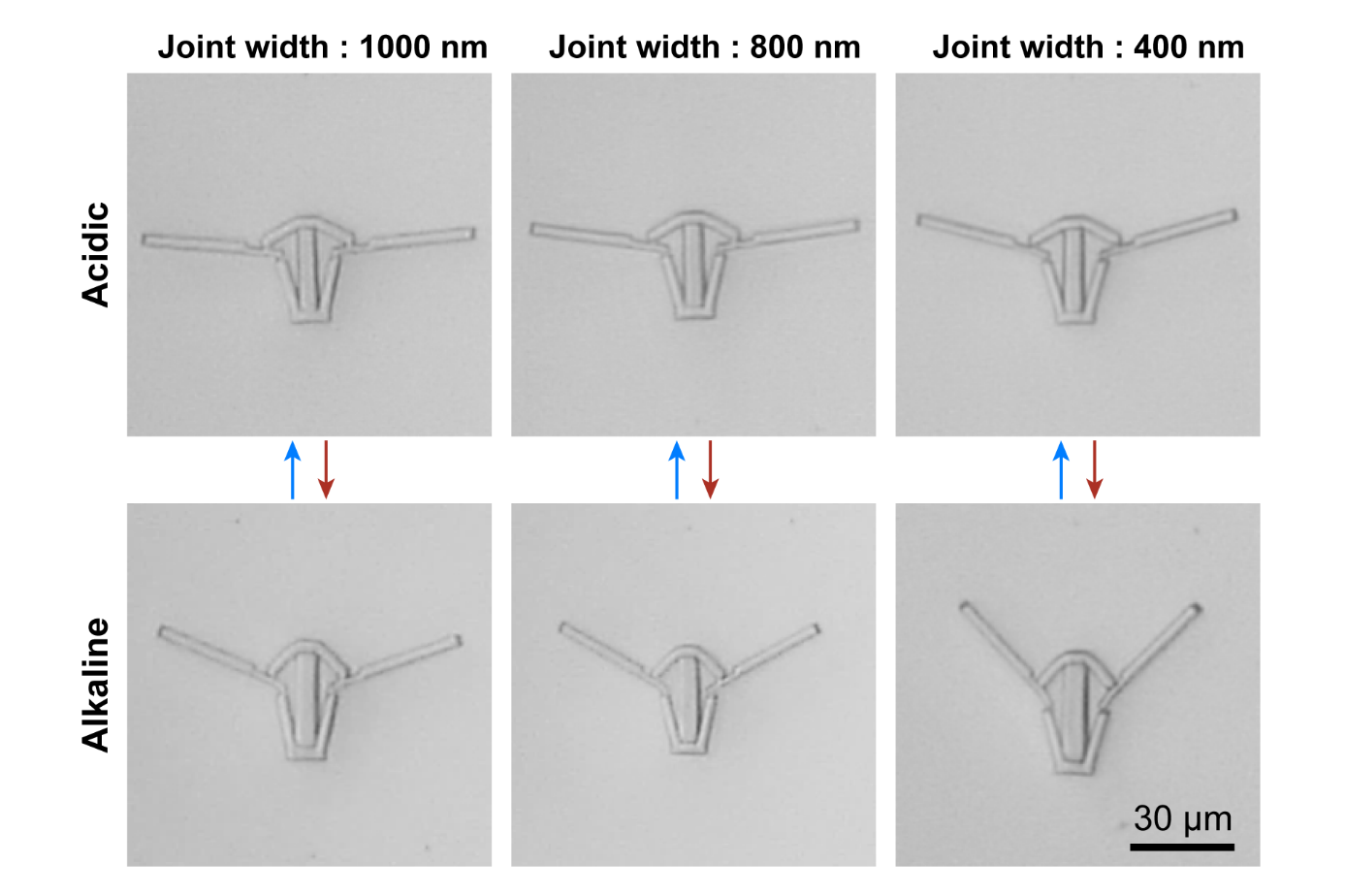


**Figure S3.** Folding deformation of microhinged actuators with different joints width (400 nm, 800 nm, 1000 nm).


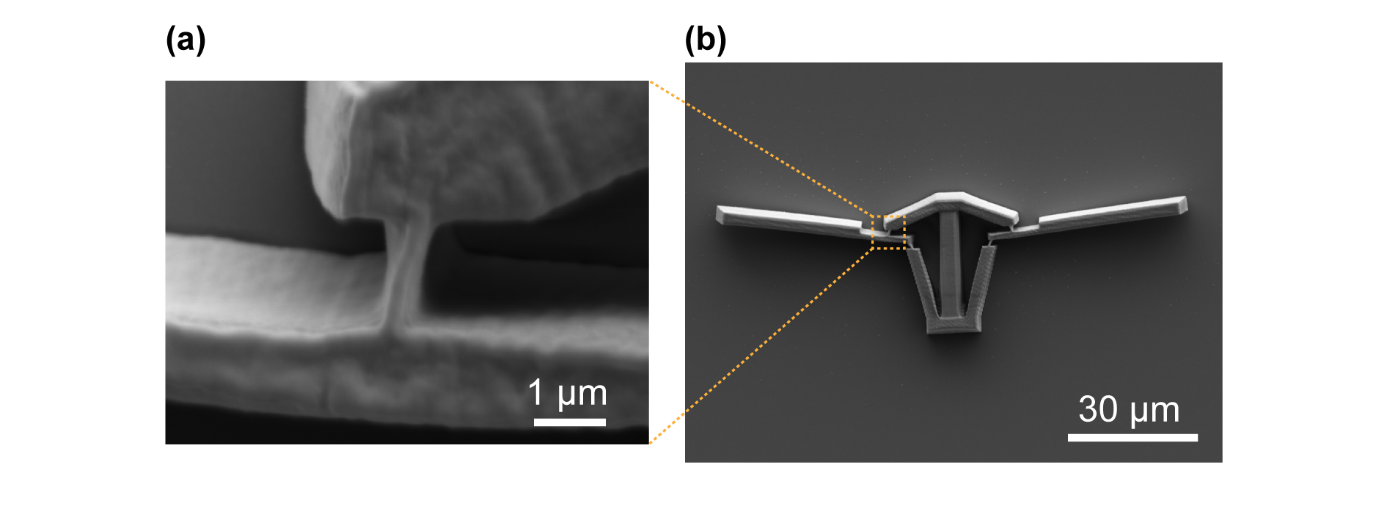


**Figure S4.** SEM images of microhinged actuator with 400 nm joint width. a) Close-up SEM image of thin joint. Scale bar: 1.5 μm. b) SEM image of initial microhinged actuator.


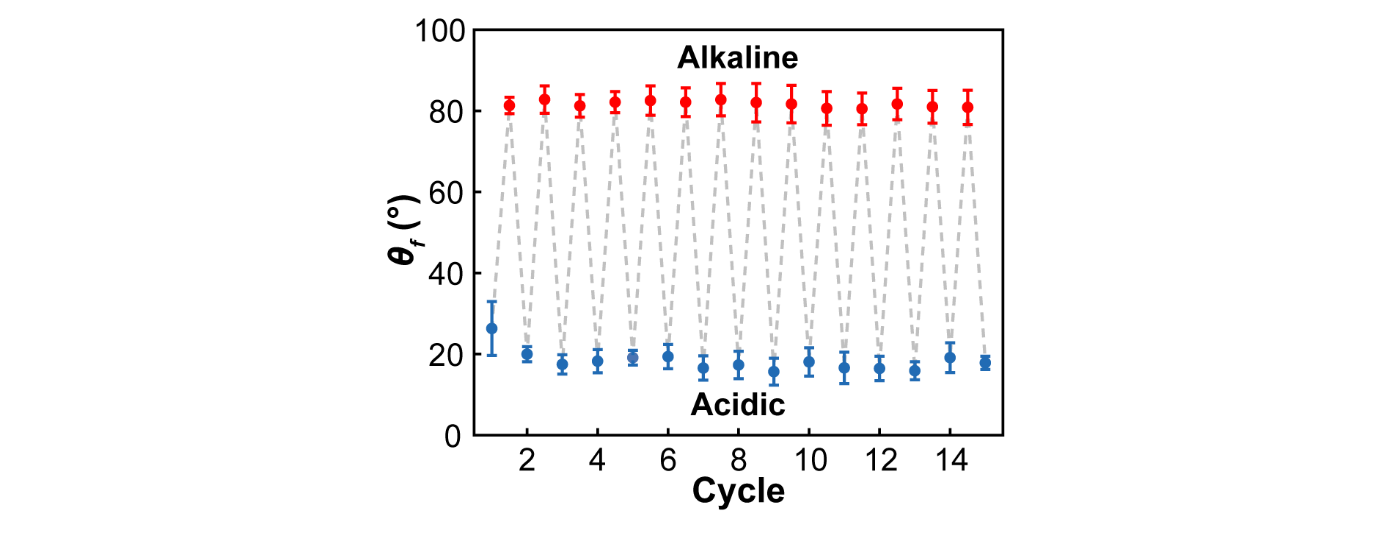


**Figure S5.** The performance of repeat deformation of the microhinged actuators.


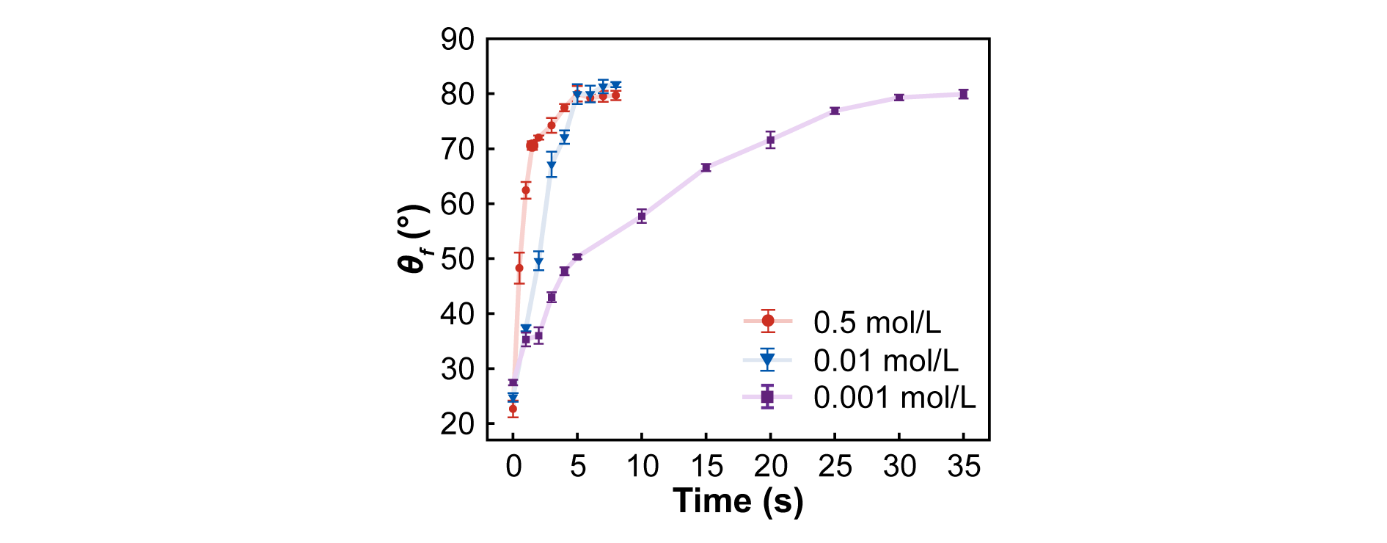


**Figure S6.** The folding angles of microhinged actuator at different time points after dropping different concentrations of NaOH solution.


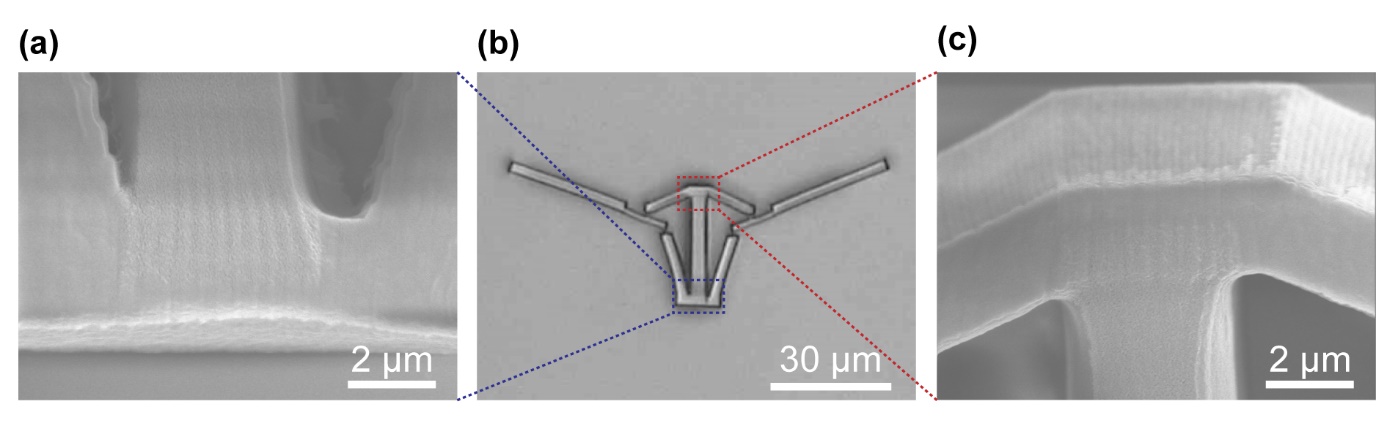


**Figure S7.** a,c) Close-up SEM image of the two-material interfaces. b) Optical images of the microhinged actuator.


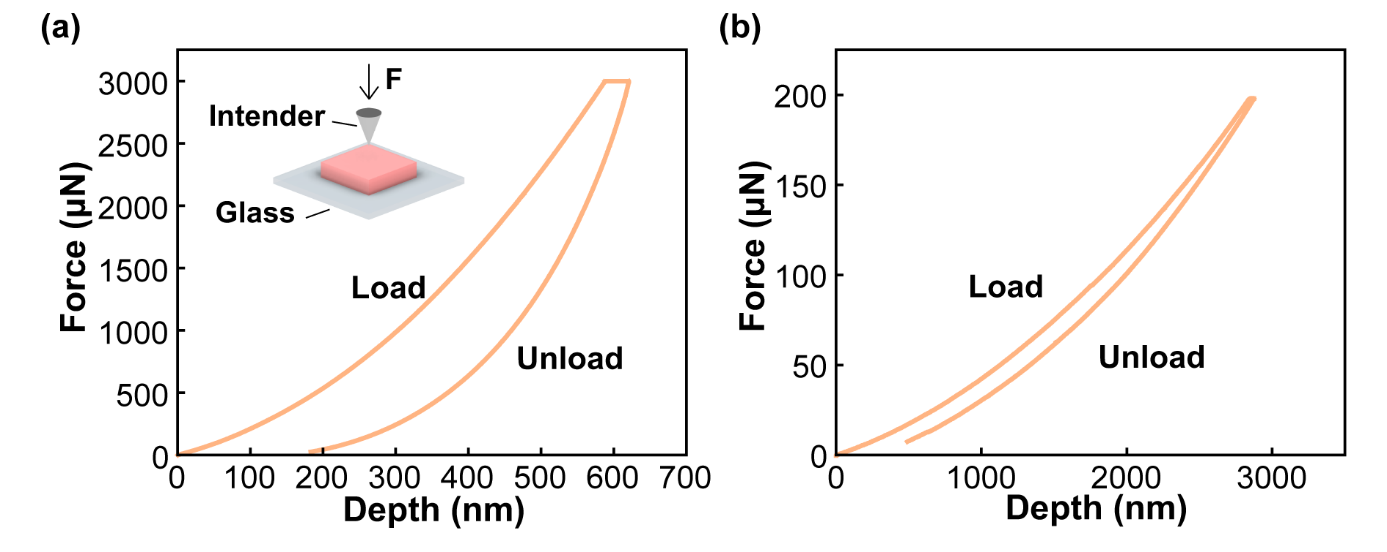


**Figure S8.** Representative force-depth curves of the a) IP-L sample and b) hydrogel sample during nanoindentation tests.


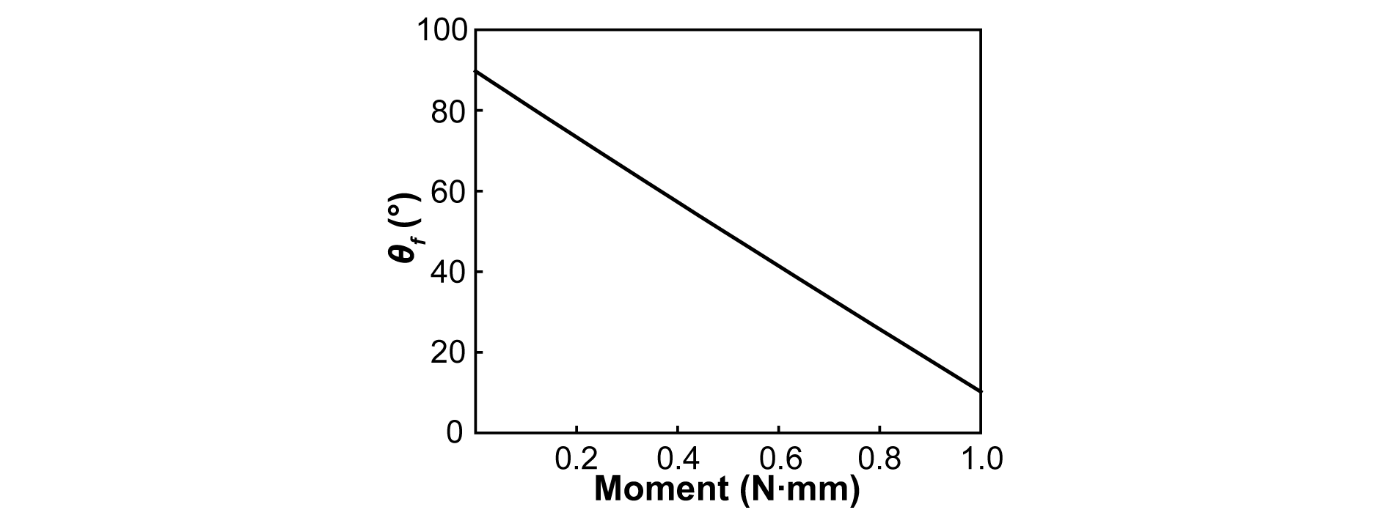


**Figure S9.** The moment-folding angle curve of the initial microhinged actuator.

References

[1] Oliver W C, Pharr G M. *J. Mater. Res*. **2004**, *19*, 3-20.

[2] L. L. Howell, A. Midha, *J. Mech. Des.* **1995**, *117*, 156-165.

[3] L. L. Howell, *Compliant Mechanisms*, Springer, New York, **2013**.

[4] D. Jin, Q. Chen, T. Y. Huang, J. Huang, L. Zhang, H. Duan, *Mater. Today*. **2020**, *32*, 19–25.

[5] T. Y. Huang, H. W. Huang, D. D. Jin, Q. Y. Chen, J. Y. Huang, L. Zhang, H. L. Duan, *Sci. Adv.* **2020**, *6*, eaav8219.

[6] Q. Y. Chen, T. -Y. Huang, P. Y. Lv, J. Y. Huang, H. L. Duan, *Adv. Intell. Syst.* **2021**, 2000232.

**Supplementary Movies**

**Supplementary Movie 1.** Shape-morphing behaviors of the initial, multi-orientation and multi-DOF microhinged actuators.

**Supplementary Movie 2.** Shape-morphing behavior of the in-plane micro-kirigami.

**Supplementary Movie 3.** Shape-morphing behavior of the out-of-plane micro-kirigami.

**Supplementary Movie 4.** Shape-morphing behavior of the 2D micro-network.

**Supplementary Movie 5.** Shape-morphing behavior of the 3D micro-network.

**Supplementary Movie 6.** Shape-morphing behavior of the micro-kirigami unit with photonic structures.
